# Errors in translational decoding: tRNA wobbling or misincorporation?

**DOI:** 10.1371/journal.pgen.1008017

**Published:** 2019-03-28

**Authors:** Xumin Ou, Jingyu Cao, Anchun Cheng, Maikel P. Peppelenbosch, Qiuwei Pan

**Affiliations:** 1 Institute of Preventive Veterinary Medicine, Sichuan Agricultural University, Chengdu, Sichuan, China; 2 Department of Gastroenterology and Hepatology, Erasmus MC-University Medical Center, Rotterdam, the Netherlands; 3 Key Laboratory of Animal Disease and Human Health of Sichuan Province, Sichuan Agricultural University, Chengdu, Sichuan, China; 4 Research Center of Avian Diseases, College of Veterinary Medicine, Sichuan Agricultural University, Chengdu, Sichuan, China; University of Maryland Medical School, UNITED STATES

## Abstract

As the central dogma of molecular biology, genetic information flows from DNA through transcription into RNA followed by translation of the message into protein by transfer RNAs (tRNAs). However, mRNA translation is not always perfect, and errors in the amino acid composition may occur. Mistranslation is generally well tolerated, but once it reaches superphysiological levels, it can give rise to a plethora of diseases. The key causes of mistranslation are errors in translational decoding of the codons in mRNA. Such errors mainly derive from tRNA misdecoding and misacylation, especially when certain codon-paired tRNA species are missing. Substantial progress has recently been made with respect to the mechanistic basis of erroneous mRNA decoding as well as the resulting consequences for physiology and pathology. Here, we aim to review this progress with emphasis on viral evolution and cancer development.

## Introduction

In all living organisms, DNA is transcribed into RNA, and RNA is translated into protein. The latter process is executed by the ribosome, which constitutes the translation machinery that produces the cellular proteome by decoding mRNAs. Deciphering mRNA codons by transfer RNAs (tRNAs) in the ribosome involves Watson-Crick base pairing [1]. However, the translation machinery is not always perfect, and errors in the amino acid composition may occur [2–5]. The general error rates of genomic replication (about 10^−8^) are estimated to be approximately 10,000-fold lower than those of protein synthesis (about 10^−4^), and thus in most instances mRNA translation is the key process contributing to inaccuracy of the cellular proteome [6]. The discrepancy between error rates in DNA replication and mRNA translation may partially relate to the fact that DNA replication occurs at the level of individual nucleotides (involving 4^1^ = 4 possible permutations), whereas the translation machinery interprets mRNA codons in triplets (involving 4^3^ = 64 possible permutations) [7].

In the canonical interpretation, 61 aminoacyl-tRNAs and 3 suppress tRNAs decode 64 triplet codons that specify 20 amino acids [1]. The resulting redundancies in the genetic code attribute to synonymous codons, which involve wobbling at position 3. For each amino acid, the number of codon usage varies from two to six according to codon degeneracy. In parallel, the numbers of certain amino acid–specified tRNAs (based on recognition of anticodons) also vary from two to six box tRNA sets. Translational decoding of the mRNA codons is constrained by factors during codon–anticodon recognition and often constitutes the rate-limiting step during protein synthesis. Besides the abundance of tRNA species, mRNA translation is regulated by nearly 100 epigenetic tRNA modifications, especially at the wobble position [8, 9]. The efficiency of mRNA decoding machinery is also essentially regulated by codon usage bias that is distinguished by over- or underrepresented synonymous codons [10, 11]. Accordingly, optimizing of tRNA wobble and codon usage in mRNA can substantially enhance translation efficiency and accuracy [10–12].

**Fig 1:**
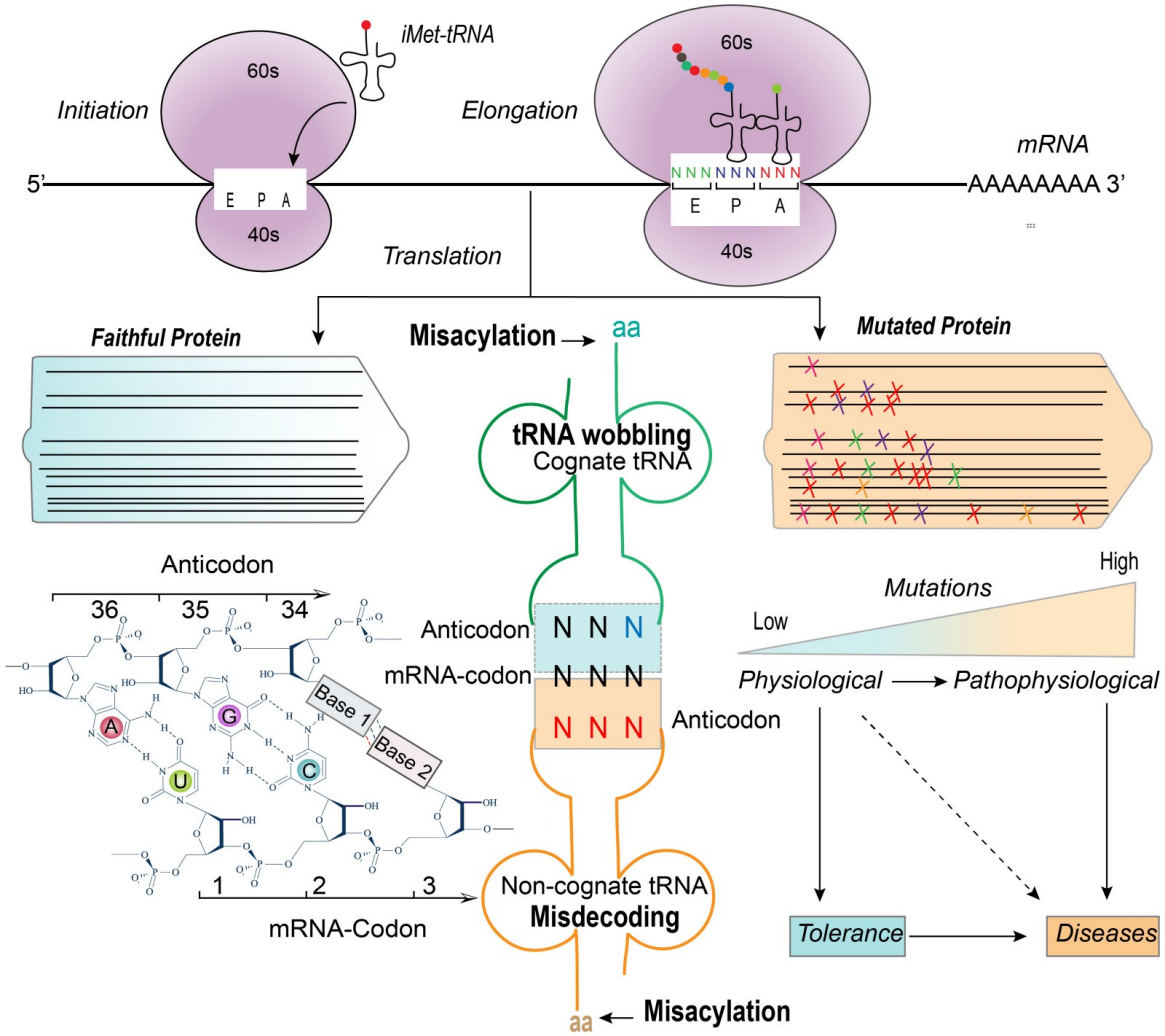
tRNA decoder regulates error ratio in translation decoding. In physiological conditions, errors in mRNA translation may occur but are generally well tolerated. However, the frequency of errors is dramatically increased in response to stresses. When amino acid misincorporation reaches intolerable levels, this contributes to dysfunction of cellular physiology and may cause pathogenesis. In general, the error ratio in translation decoding primarily depends on tRNA wobbling (cognate) and misdecoding (noncognate) as well as misacylation of tRNAs. aa, amino acid; iMet-tRNA, initiator tRNA Methionine; tRNA, transfer RNA.

Nevertheless, mistranslation universally occurs. Pre- or post-mRNA translation may indirectly introduce errors of protein synthesis during transcription and posttranslational processing [13]. However, the translation machinery can directly contribute to mistranslation by tRNA misdecoding (leading to misincorporation or stop-codon readthrough), tRNA misacylation (leading to wrong tRNA–amino acid coupling), codon reassignment or ribosomal translocation-provoked frameshifts (Fig 1) [13]. It is becoming increasingly clear that such mistranslation has consequences on the pathophysiology of a variety of diseases (Fig 1) (Table 1), including multiple sclerosis, neurodegeneration, mitochondrial myopathy, encephalopathy, lactic acidosis, stroke-like episodes, Parkinson's disease, and cancer [14–19]. In this review, we aim to describe the key mechanisms that underlie mistranslation and illustrate potential implications using viral evolution and carcinogenesis as examples.

**Table 1:** The types and outcome of errors in translation machinery.

Organism	Error rates	Outcome	Cause	Mistranslation	tRNAs	Reference
*E*. *coli*	10%	Tolerance	Misacylation	Cys→Pro Ser→Thr Glu→Gln Asp→Asn	tRNA^Pro^ tRNA^Thr^ tRNA^Gln^ tRNA^Asn^	[41]
*Drosophila*	Approximately 10%–60%	Cell death	Misacylation	Tyr→Phe	tRNA^Phe^	[87]
Yeast	Approximately 6%	Stress nonsensitive	Misacylation	Pro→Ala	tRNA^Pro^(U/A)GG	[88]
HeLa cells	Approximately 5%	Alleviate oxidative stress	Misacylation	Glu→Met	tRNA^Glu^	[45]
CHO cells	Approximately 0.7%	Without changing in cellular viability	Misacylation	Tyr→Phe	tRNA^Tyr^	[89]
Mouse	Approximately 40%–50%	Neurodegeneration	Misacylation	Gly→Ala Ser→Ala	tRNA^Ala^	[15]
Ciliates	?	Genetic code evolution	Misdecoding	Gln→UAA/UAG Trp→UGA	tRNA^glu^ tRNA^Trp^	[34]
Yeast	Approximately 45.5%–54%^①^ Approximately 0.5%^②^ Approximately 7%–86%^③^	Stop-codon reassignments	Misdecoding	Gln/Tyr→UAA(Stop)^①^ Lys→UAG(Stop)^②^ Arg/Cys/Trp→UGA(Stop)^③^	tRNA^Glu/Tyr^ tRNA^Lys^ tRNA^Trp/Arg/Cys^	[2]
Mice	Approximately 200%–400%	Tumor growth	Misreading	Ser→Ala	tRNA^Ser^	[18]
Mycobacterial	Approximately 0.2%^①^ Approximately 0.8%^②^	Rifampicin resistance	Misincorporation	Gln→Glu^①^ Asn→Asp^②^	tRNA^Glu①^ tRNA^Asp②^	[43]
Human	?	Multiple sclerosis	Misincorporation	Aze→Pro	Likely tRNA^pro^	[14]
Human	?	Mitochondrial disease	Wobble modification	Leu(UUG) reduced translation	tRNA^leu^	[16]
Plant	?	Antibiotic sensitivity	Wobble and superwobbling	?	All codons with pyrimidines at wobble sites	[21]
Human	Approximately 20%–80%	Proper translation	Modified wobble	Met→Ile (AUA)	tRNA^Met^_f5CAU_	[90]
Yeast	Approximately 97.2%	Spontaneous	Codon reassignment	Ala→Leu (CUG)	tRNA^Ala^-CAG	[91]
Human	?	Type 1 diabetes	Frameshift	?	?	[92]

The questions marks indicate unknown data of provided examples. The numbers in circles specify types of amino acid substitute and corresponding data at each row.

Abbreviations: Aze, azetidine-2-carboxylic acid; CHO, Chinese hamster ovary; tRNA, transfer RNA.

## tRNA wobbling compensates for missing tRNA species

**Fig 2:**
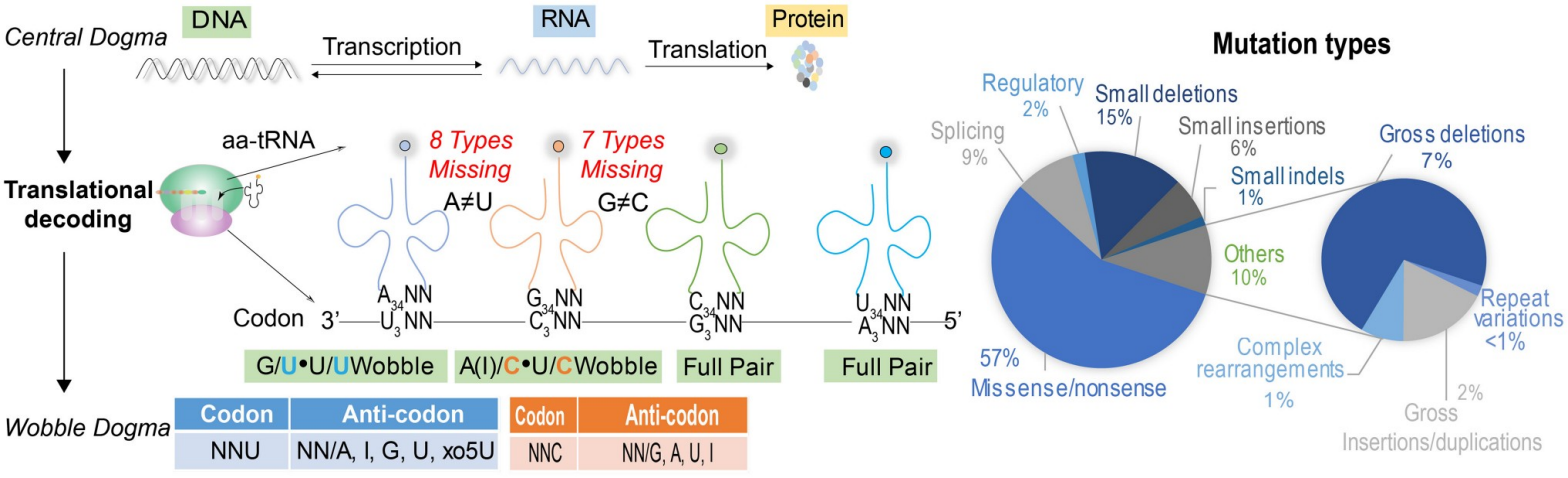
tRNA wobbling increases the risk of mistranslation. In the central dogma, DNA transcribes RNA and RNA translates protein. In the human mutation database, the major (57%) mutation types are missense/nonsense (the right panel) that reflect the consequence of DNA errors at genomic level. Ribosome, as the translation machinery, essentially transduces genetic code to functional protein performed by aa-tRNAs. In human genome, 15 out of 64 tRNA types are actually missing partially because of low confidence (score < 50), including eight tRNA^ANN^ and seven tRNA^GNN^. Because of these missing tRNAs and the expanding wobble rules, the mRNA codon can be decoded by cognate or noncognate tRNAs, leading to modulation of translation efficiency and misincorporation (the left panel). At the bottom, the revised wobble rules and the consequent wobble types are listed according to the wobble position 3 of triplet codon. As for eight tRNA^ANN^, NNU codons will be decoded by NNG or NNU anticodon of tRNAs. As for seven tRNA^GNN^, NNC codons will be decoded by NNA(I) or NNU anticodon of tRNAs. For specific missing tRNAs, the consequent wobble (tRNA wobbling or misdecoding) are detailed in Fig 3. aa-tRNA, aminoacyl-tRNA; tRNA, transfer RNA.

In the ribosome, tRNAs detect appropriate mRNA codons using the anticodon loop and transfer proper amino acids to polypeptides. However, the number of obligatory tRNA species (based on anticodons) for mRNA translation is substantially smaller than the theoretically required 64 species necessary for full codon matching [1]. Life solves this problem by allowing wobbling or superwobbling (also known as the “four-way wobbling”), thus allowing fewer tRNA species to translate all mRNA codons (Table 1) [20–22]. In the human genome, there are approximately 10-fold excess of tRNA gene copies as compared to the number of possible codons (613 versus 64) [23, 24]. Nevertheless, the recently released GtRNAdb 2.0 database indicates that 15 out of theoretically necessary 64 tRNA species are missing, partially because of low confidence (scores < 50), including eight tRNA^A34NN^ and seven tRNA^G34NN^ (Fig 2) [23].

**Fig 3:**
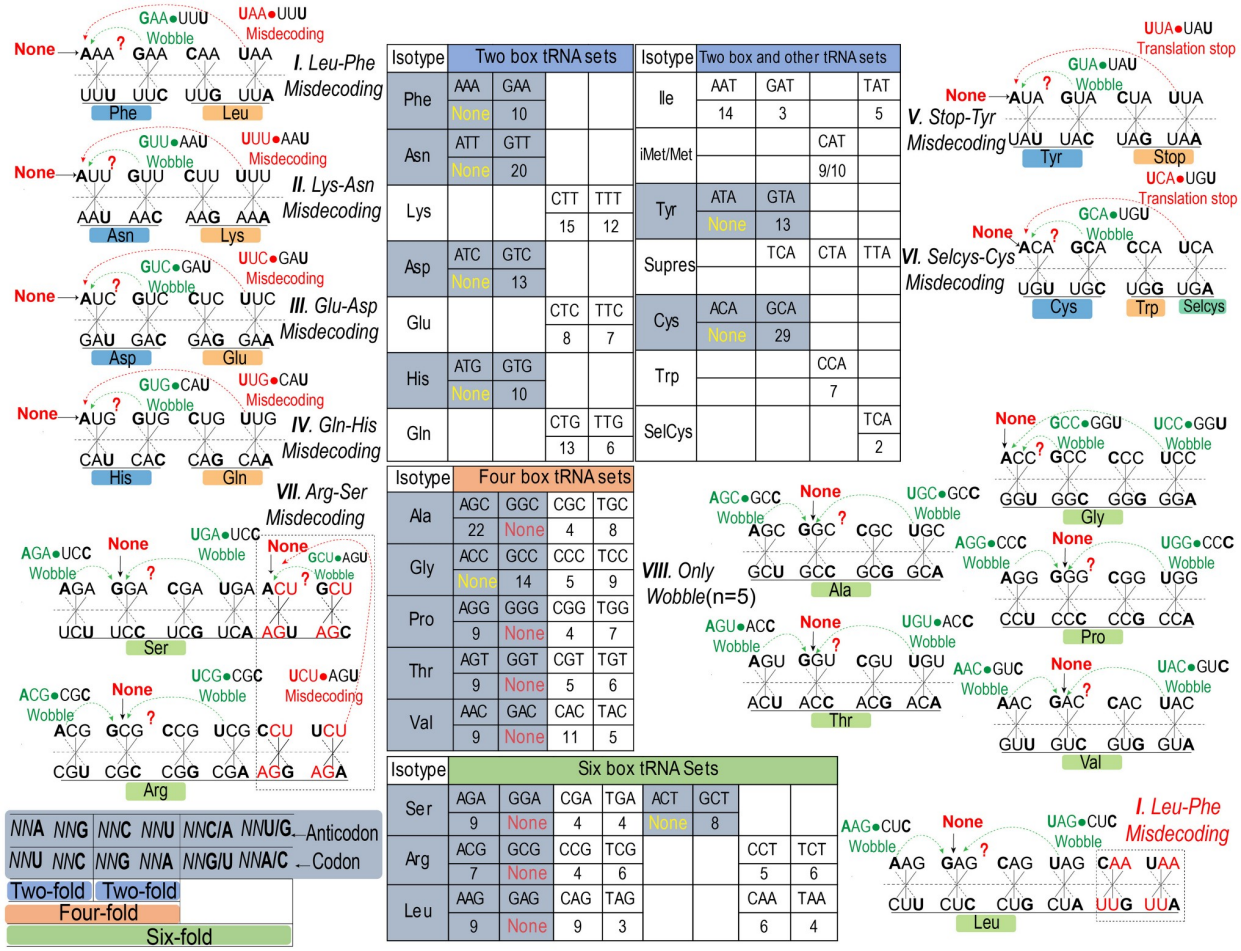
Errors in translation decoding are regulated by tRNA wobbling at all three codon positions. Sixty-four tRNA sets are summarized and specified in parallel with codon degeneracy (left bottom and central). Faithful or misincorporated protein can result from decoding by cognate or near-cognate tRNA at position 3. For eight missing tRNA^ANN^ (yellow text), NNU•tRNA^UNN^ wobble-dependent misdecoding by near-cognate tRNAs mainly occurs at the two box (I–VI) and six box tRNA sets (arginine-serine misincorporation) (VII). For seven missing tRNA^GNN^ (red text), NNC codons will be decoded by cognate tRNAs without amino acid misincorporation (VIII) because they happen at the four and six box tRNA sets. Since leucine and phenylalanine share UUN codon, leucine-phenylalanine misincorporation may occur across the six and two box tRNA sets. Besides wobbling at position 3, mRNA codons can be falsely decoded by “far-cognate” tRNA at position 1 and 2 (in the text). Missing tRNAs are indicated as question mark. Individual wobble and misdecoding are labeled as green and red text, respectively. tRNA, transfer RNA.

How to decode these codons without fully paired tRNAs remains an intriguing question. Because of wobbling and superwobbling, it is possible to use 32 tRNA species for decoding all 64 possible codons [1, 22]. In plastid genomes, even 25 tRNA species suffice protein biosynthesis by “four-way wobbling” [21]. tRNA species with an unmodified U at wobble site can decode all four triplets (NN/A, G, C, and U). This relaxed wobble has been identified in *Mycoplasma* spp. and particular organelles, including mitochondria and, as mentioned, in plastids [21, 25, 26]. Therefore, to decode those unpaired codons, cognate or noncognate tRNAs are forced to wobble at position 3 of the codons by wobbling or superwobbling (Fig 3).

## Decoding unpaired codons by excessive tRNA wobbling

Though tRNA wobbling enables translation compatibility, this also increases the probability of misdecoding by noncognate tRNAs. Among the eight missing species of tRNA^ANN^, the NNU codons likely only pair with tRNA^GNN^, tRNA^UNN^, and tRNA^INN^ as dictated by the revised wobble rules (Table 2) (Fig 2), because the tRNA^ANN^ is missing, and so does tRNA^INN^ (leading to wobbling with either adenine, cytosine, or uridine), as conversion of tRNA^ANN^ to tRNA^INN^ is catalyzed by the tRNA-dependent adenosine deaminases 2 (ADAT_2_) [27]. Specifically, if NNU codons pair with tRNA^GNN^, it will lead to a G•U wobble pair without concomitant amino acid misincorporation, as the same amino acid is coded by NNU and NNC (tRNA^GNN^) codons (Fig 3). If NNU codons pair with tRNA^UNN^, however, the resultant U•U pair will cause amino acid misincorporation. NNU and NNA (tRNA^UNN^) code for different amino acids at the 2-fold degenerate codon box, resulting in leucine→phenylalanine, lysine→asparagine, glutamic acid→aspartic acid, glutamine→histidine, Stop→tyrosine, and selenocysteine→cysteine misdecoding (Fig 3). According to the original wobble hypothesis of Francis Crick, the codons decoded by the two box tRNA sets must distinguish either NNU/C or NNA/G [1]. However, based on the revised wobble rules, NNU•tRNA^UNN^-mediated decoding is at bay with Crick’s assumption and might lead to misincorporation of amino acids. Leucine→phenylalanine, lysine→asparagine, and glutamine→histidine misincorporations have been reported to occur in bacterial and mammalian cells when such cells suffer from phenylalanine, asparagine, and histidine starvation, respectively [28–30]. Misreading of codons by the “Two-out-of-three” hypothesis, which entails that the first two nucleotides in each codon are essential for anticodon recognition, has been suggested to pose a threat to translation fidelity [31]. This type of misreading may occur in those 2-fold degenerate codons as uniquely discriminated by wobble bases. It has been experimentally proven that tRNA superwobbling suffices to decode all four triplets of 4-fold degenerate codons in plastids [21, 22]. Such superwobbling may allow the 2-fold degenerate codons to cross-decode by NNU•tRNA^UNN^-mediated decoding (Fig 3).

**Table 2:** Revised wobble rules.

Codon (XXN_3_)	Anticodon (N_34_XX)
A	U, A, I, xo^5^U, xm^5^s^2^U, xm^5^Um, Um, xm^5^Um, k^2^C
U	A, I, G, U, xo^5^U
G	C, A, U, xo^5^U, xm^5^s^2^U, xm^5^Um, Um, xm^5^Um, m^5^C
C	G, A, U, I

With respect to the seven missing species of tRNA^GNN^, the NNC codons are expected to pair with tRNA^ANN^, tRNA^UNN^, and tRNA^INN^ (Fig 2) (Table 2). All missing tRNA^GNN^ can be decoded through wobble pairing without accompanying misincorporation of amino acids (Fig 3) because they occur in 4- or 6-fold degenerate tRNA boxes for which cognate tRNAs are available (Fig 3). However, misincorporation of amino acid may occur if NNC codons are misdecoded by noncognate tRNA^UNN^, in which the amount of tRNA^GNN^ is not limiting, because NNC and NNA (tRNA^UNN^) code for different amino acids in twice-degenerated codons (Fig 3). Furthermore, codon UA**U** (tyrosine)-UA**A** (Stop) mismatch will truncate the elongation process of nascent peptide (Fig 3). Conversely, if the stop codon (UGA) is mismatched by a Selcys-tRNA^UCA^, this will lead to an excessively translation elongation (Fig 3). Arginine and serine share an AG**N** wobble (AG**A** and AG**G** for arginine; AG**U** and AG**C** for serine), and this predisposes organisms to a potential arginine→serine misincorporation (Fig 3). Such arginine→serine misincorporation affects the quality of therapeutic antibody production by Chinese hamster ovary cells, illustrating the relevance of mRNA misdecoding [32]. In conclusion, unpaired codons are likely to be misdecoded by noncognate tRNAs because of excessive tRNA wobbling, raising questions as to the consequences of such misdecoding for living organisms [33].

## tRNA wobbling at three codon positions compromises the fidelity of the translation decoder

Nonsense translation, so-called stop-codon readthrough, can result from aberrant decoding of stop codons by noncognate aminoacyl-tRNAs (examples are Gln[**C**AG/**C**AA], Tyr[UA**U**/UA**C**], and Lys[**A**AG/**A**AA] for the UAA and UAG stop codons respectively; Trp[UG**G**], Arg[**A**GA], and Cys[UG**U**/UG**C**] for the UGA stop codon) [2, 33]. The occurrence of such readthrough highlights the possibility of position 3 and 1 wobbling in translational machinery and provides an indication as to how common translational misdecoding in living organisms is. In ciliates, ribosome profiling has demonstrated that all three stop codons can be misdecoded, whereas rates of such miscoding depend on the position within mRNA molecule (coding region or the end) [34]. Position 1 wobbling occurs not only in stop codons but also in sense codons, such as the misreading of arginine **C**GU/**C**GC codons as cysteine **U**GU/**U**GC codons [35, 36]. By using the prokaryote ortholog of elongation factor Tu (EF-Tu) for targeted mass spectrometry, it has been reported that even position 2 can be misdecoded by noncognate tRNAs, as illustrated by the detection of the arginine C**G**U codon misdecoded by tRNA^G**A**G^-Leu [5]. Thus, substantial misdecoding at all three positions is possible [2, 5] (Table 1). This consequently compromises the fidelity of the translation decoder.

It has been reported that G•T mismatching occurs in both DNA and RNA duplex following tautomerization and ionization, and this plays important roles in replication and translation errors [37, 38]. The Watson-Crick-like mismatch can evade fidelity checkpoints and appears to occur with probabilities (10^−3^ to 10^−5^) that strongly imply a universal role of this mismatch in translation errors [38]. The rG•rT mismatch at position 3 may not lead to mistranslation in decoding center, because NNU and NNC (rG•rC/rU) code for the same amino acids in such twice-degenerated codons, and the same holds true for NNG and NNA (rU•rA/rG) (Fig 3). However, more mistranslation results if rG•rT mismatch takes place at position 1 and 2 [5, 35, 36]. Hence, in toto a picture emerges—that amino acid misincorporation in the nascent peptide chain is prone to occur mainly because of the absence of fully Watson-Crick pairing tRNAs and by excessive wobbling at all three codon positions [5].

## Quality control of the translation machinery

**Fig 4:**
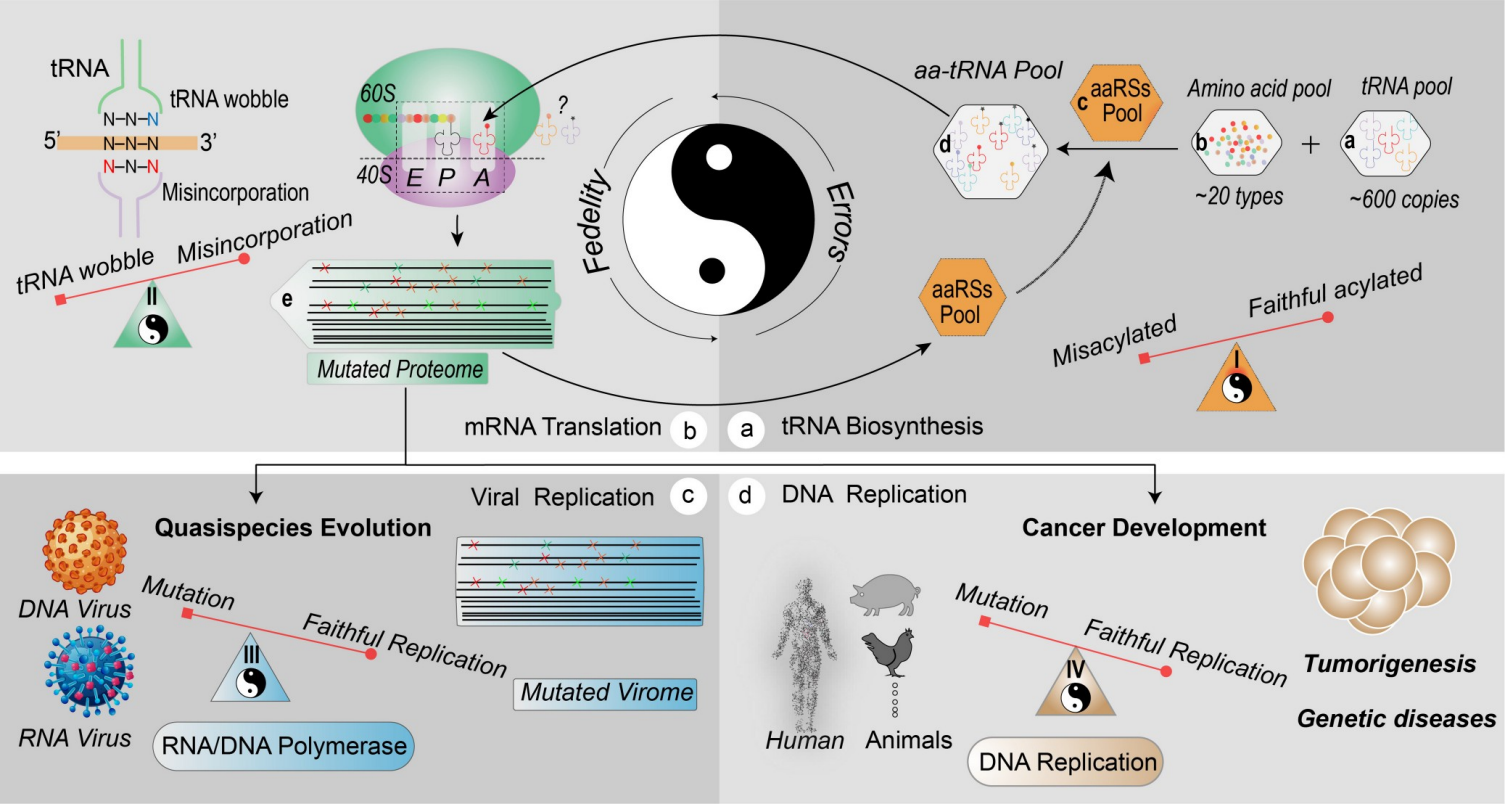
Fidelity and errors of translation decoding and the implications in viral evolution and cancer development. (a) aa-tRNAs are synthesized by sampling from the amino acid pool and tRNA pool and require catalysis by aaRSs. This process may accidently introduce misacylated aa-tRNAs, because the types of tRNAs and amino acids are difficult to be distinguished by involved aminoacyl synthetase because of analogous structures. (b) During elongation, tRNA wobbling will increase translation efficiency. Misincorporation can also be introduced because of tRNA misdecoding (amino acid misincorporation caused by excessive wobble decoding), especially when certain codon-paired tRNA species are missing. Finally, the fidelity of translation machinery will be impaired and produce mutated proteome, including RNA and DNA polymerases, aaRSs, and accessories. (c) Mistranslation of RdRP in RNA viruses will augment generation of a mutated virome (quasispecies) and facilitate viral evolution and adaption. (d) Similarly, mistranslation of cellular DNA replication-related enzymes and relative proteins amplifies mutagenesis in the genome and contributes to cancer development. aaRS, aminoacyl-tRNA synthetase; aa-tRNA, aminoacyl-tRNA; RdRP, RNA-dependent RNA polymerase; tRNA, transfer RNA.

Faithful translation of the mRNA codons into protein is essential for cellular physiology. The fidelity of the translation machinery firstly depends on the specific coupling of amino acids to their cognate tRNA species, which is catalyzed by aminoacyl-tRNA synthetases (aaRSs) (Fig 4a and 4b). aaRS is capable of discriminating its cognate substrates from structurally analogous tRNAs and amino acids [39]. Subsequently, eukaryotic elongation factor 1A (eEF-1A) or prokaryotic EF-Tu delivers the aminoacyl-tRNA to the ribosome A site for elongation of nascent peptide chain after proper codon–anticodon recognition [40]. Thus, aaRSs are cardinal in protecting protein synthesis against misacylation [39], but their specificity is not absolute. For instance, in *E*. *coli*, four types of misacylated-tRNA—including Cys-tRNA^Pro^, Ser-tRNA^Thr^, Glu-tRNA^Gln^, and Asp-tRNA^Asn^—do not evoke a correctional reaction [41]. In both mice and bacteria, serine is prone to be misacylated by alanyl-tRNA synthetases (AlaRSs) [42]. In mycobacteria, an increase in the substitution of glutamic acid→glutamine and aspartic acid→asparagine by translational misincorporation has been linked to phenotypic resistance to rifampicin treatment [43]. Thus, beneficial mistranslation in both prokaryotes and eukaryotes may exist and improve their survival or facilitate drug resistance [43–45]. Apart from misdecoding, misacylation of amino acids to tRNA molecules is another important source of mistranslated proteins, despite the presence of mechanisms preventing such events.

How could tRNA wobbling guarantee faithful decoding by the codon–anticodon duplex? During elongation, eEF-1A or EF-Tu delivers amino acid–coupled tRNA to the ribosome A site [40]. Subsequently, the ribosome rechecks the codon–anticodon duplex that involves the highly conserved G530, A1492, and A1493 of 16S RNA via stabilization of the first two Watson-Crick pairs of the duplex [31, 46]. A correct confirmation of the codon–anticodon duplex will induce a conformational domain closure in the ribosome and result in the formation of the appropriate peptide bond and elongate the nascent protein [47]. Analysis of X-ray structures suggests that the positions 1 and 2 of the A codon are obligatory Watson-Crick base pairs. In prokaryotes, when U•G and G•U wobbles at the first or second codon–anticodon position, the decoding center forces this pair to adopt the geometry close to that of a canonical C•G pair [40]. Using nuclear magnetic resonance (NMR) relaxation dispersion, it has recently been revealed that dG•dT misincorporation during replication is likely mediated via tautomerization and ionization [37]. As discussed, these Watson-Crick-like mismatches may further contribute to tRNA wobbling and consequently misdecoding [5]. Although the hydrogen bond is the major force to form codon–anticodon pairs [1], the van der Waals forces, steric complementarity, and shape acceptance may concurrently contribute to the codon–anticodon recognition essentially for quality control [3, 40].

## mRNA mistranslation in physiology

The integrity of mRNA translation sustains essential cellular physiology in all domains of life. Low level of mistranslation, however, is well tolerated and even contributes to stress responses, as it creates a degree of diversity in the proteome (also known as “statistical proteome”) [4]. Yeasts engineered to misincorporate serine at leucine CUG codon initially lose fitness but quickly adapt by promoting the evolution of genome architecture [48]. Experiments employing misacylated aminoacyl-tRNAs show that up to 10% of overall mistranslation in *E*. *co*li does not compromise physiology of this organism and is even compatible with bacterial proliferation [41]. aaRSs of mycoplasma with mutations in the editing domain provoke misacylation tRNAs with highly similar amino acids that contribute to antigen diversity as to escape host immune defenses [49]. In mammalian cells, up to 10-fold methionyl-misacylation to non–methionine-tRNAs will protect against reactive oxygen species (ROS)-mediated damage when cells undergo oxidative stress, such as exposure to viral infections, Toll-like receptor ligands, or xenobiotics [45].

Rates of mistranslation vary dramatically between organisms and different environmental conditions (Table 1). An overall amino acid misincorporation rate of approximately 3‰–5‰ during translation is regarded as compatible with normal physiology [50, 51]. In contrast, exceeding 1% misincorporation is usually deleterious and may provoke pathogenesis [43]. For example, the 50% tRNA^Ala^ mischarging with serine residues by an editing-defective AlaRS is associated with neurodegeneration [15]. In addition, defective AlaRS is also related to cardioproteinopathy [52]. However, the capacity of organisms to deal with mistranslation appears diverse, and subphysiological mistranslation is tolerant and even beneficial.

## Errors of translation and viral evolution

Viral genomes are dynamically mutated with frequent emergence of new quasispecies. The spectrum for the hypermutation of viral genomes, sometimes denominate as mutant clouds [53]. Mutation rates at genomic level (substitutions per nucleotide per cell infection [s/n/c]) range from 10^−8^ to 10^−6^ s/n/c for DNA viruses and from 10^−6^ to 10^−4^ s/n/c for RNA viruses [54]. Apparently, there is an error threshold to constrain viral evolution dependent on the genome size and permutations of errors [55]. Within the virome, RNA viruses in particular mutate tremendously as a consequence of RNA-dependent RNA polymerases (RdRPs) being error-prone. For instance, the mutation rate of RdRPs that mediate poliovirus and foot-and-mouth disease virus (FMDV) replication can further expand or reduce the quasispecies diversity by regulation of replication fidelity [56, 57]. The consequences of the mutations highly depend on both the position and properties of the affected amino acid residues. To take FMDV as an example, a W237F mutation but not a W237I mutation in the polymerase leads to a high fidelity and thus contributes to the subsequent mutation rates [57].

Little is known of the consequences of an error-prone translation machinery on viral evolution. As discussed, erroneous protein synthesis is prone to occur especially when cells suffer cellular stresses like viral infection. In this situation, the viral RNA is possibly mistranslated during the inaccurate translation [6]. Several types of errors in the translational machinery have been linked to viral adaptability. An example is the apparent selective pressure exerted on fungal mitovirus to exclude UGA (tryptophan) codons from its coding sequence because of the lack of fidelity of decoding this codon by the host mitochondrion [58]. When organisms are recoded to obtain nonassigned codons, compensatory mechanisms emerge, including frameshifts and stop-codon readthrough [59]. In yeast, mistranslation has been demonstrated to provoke evolution of genomic architecture [48]. Hence, genomic mutagenesis is substantially associated with mistranslation that promotes the likelihood of evolution [4]. It is well possible that viruses also utilize mistranslated genome-copying machinery (e.g., RdRP) for viral evolution. Besides the classical mutations inherited from error-prone replication at the genomic level, we propose that mistranslation may generate additional RdRP mutants at the protein level that are not inheritable. Except for negative-strand RNA viruses, there is no RdRP incorporated in the virion. Therefore, upon infection, translation of the viral genome is invariably ahead of its replication. Thus, when an RNA virus releases its genome into the host cell after uncoating, errors in RdRP may be accidently introduced by mistranslation [58, 59]. The resulting mixture of wild-type and mutated RdRP enzymes initiate replication associated with a spectrum of viral quasispecies (Fig 4c). Those species that possess the best viral fitness finally survive and become dominant.

As for DNA viruses, the mechanisms driving viral mutation are more diverse and less well understood. Degradation of HIV-1 proviral DNA with G→A hypermutation has an important role in host responses to infection [60]. This sublethal mutagenesis catalyzed by cytidine deaminases in the family of apolipoprotein B RNA-editing catalytic polypeptide-like 3 (APOBEC3) can induce drug-resistant and generate immune-escape viruses [60, 61]. In hepatitis B virus–infected patients, however, such mutations may have undesired consequences with respect to the viral reverse transcriptase (e.g., the A181T and M204I mutations) and mediate adefovir resistance [62]. Analogously, it has been reported that mutations in palm, finger, and 3′-5′ exonuclease domains of herpesviruses DNA polymerase are introduced as a consequence of nucleoside analogue-based therapy [63]. Mistranslation in DNA viruses can also generate viral proteins that are more prone to provoke mutations in the viral genome, but hard data for this notion are currently still lacking.

## Errors of translation in cancer development

Malignant transformation is usually associated with accumulation of large numbers of DNA mutations. Once occurring in essential oncogenes and tumor suppressors, these are also intimately associated with cancer development and progression [64, 65]. The importance of DNA mutation-dependent alteration in protein composition is illustrated by the recent identification of approximately 3,400 driver mutations in tumor exomes [66]. In the human mutation database, 57% of the mutations are missense/nonsense (Fig 2). This reflects the major consequences of DNA errors that are driven by either DNA replication errors or environmental factors. Apart from genomic alterations, mistranslation may also be important in cancer cells. It has been reported that DNA replication errors are responsible for two-thirds of the mutations observed in 17 cancer types [67]. Hence, reduced fidelity of DNA-replicating enzymes appears more important than environmental factors for generating cancer-associated mutations. The implication of this notion is that if mistranslation of DNA-replicating enzymes reduces replication fidelity, this would be expected to further advance cancer development [68]. Of note, translation machinery is largely rewired during tumorigenesis [69]. By shaping tRNA pool to match protumorigenic mRNAs, the translation of oncogenes is facilitated to prime oncogenesis, such as highly up-regulated tRNA^Glu^-UUC and tRNA^Arg^-CCG in breast cancer [70, 71]. Moreover, mutated components of ribosome are involved in carcinogenesis as well and may foster disease by compromising the ribosome (translation fidelity) to “translate” cancer [69]. For example, missense mutations of the ribosomal protein RPS15, a component of the 40S ribosomal subunit, is involved in chronic lymphocytic leukemia [72]. How the compromised translation machinery contributes to the nature of hypermutated tumor transformation at the genomic level is an intriguing question. In analogy to RNA viruses, mistranslation of DNA polymerases and APOBEC3H in cancer may occur before genomic replication [68, 73–75]. The cellular proteome in G1 phase of cell cycle must duplicate before S phase, and the demand on the translational machinery may provoke errors with respect to mRNA decoding [76]. The ribosomal fidelity in (pre-) malignant cells may become compromised, resulting in mistranslated DNA polymerase molecules, which in turn drive further genomic instability [69, 75]. This further contributes to hypermutation and consequently tumorigenesis (Fig 4d) [70, 77]. In apparent support of this notion, mistranslation caused by serine-to-alanine misreading tRNA has been shown to promote the development of epithelial cancer in mouse models [18]. Moreover, mutated DNA polymerase ε (P286R) in mice models provokes ultra-mutagenesis that can rapidly develop into lethal cancers of diverse lineages [75].

It is important to note that N→T missense mutations are widespread in cancer [66]. This type of mutation increases translation efficiency through facilitating tRNA wobbling and superwobbling that provides the cancer cells with advantage to compete clones but will concomitantly provoke amino acids misincorporation, especially when the two box tRNA sets are involved (Fig 3). As described, epigenetic modification of tRNA (U34) further supports tumorigenesis by up-regulating U34 enzymes and enhancing codon wobble of especially tumor promoting genes, an effect that prominently involves SRY-box 9 (SOX9) and elongator complex protein 3 (Elp3) [78, 79]. A high level of the U34 enzyme promotes alternative translation and has been linked to resistance to anti-BRAF therapy through wobble decoding of hypoxia-inducible factor 1A (HIF1A) mRNA in a codon-specific manner [80]. Thus, the error-prone translation machinery appears to contribute to mutagenesis during cancer development.

Though mistranslation promotes carcinogenesis, it also offers possible targets for anticancer therapeutics. Targeting enzymes catalyzing U34 tRNA modification has been demonstrated the potential for treating melanoma [81]. Depletion of the U34 enzymes Elp3 or cytoplasmic tRNA 2-thiolation protein 1/2 (CTU1/2) provokes cell death in patient-derived BRAF^V600E^ melanoma cultures [80]. Genetic incorporation of noncanonical amino acids by decoding specific a codon is another approach [82]. Misincorporations of p-acetylphenylalanine at target codons have been explored to develop bispecific antibody-based therapy for breast cancer and acute myeloid leukemia [83, 84]. Moreover, certain mutant peptides of human tumors can serve as T-cell epitopes for immunotherapy [85]. These tumor-specific immunogens as potentially personalized vaccines have been shown to boost immune rejection to the tumors in mouse model [85, 86].

## Conclusion and perspective

mRNA mistranslation universally occurs across all living organisms. It is generally well tolerated in physiology and even helps the organism adapt and withstand cell stresses. However, excessive mistranslation is pathogenic and implicated in many diseases. Mistranslation may also provide targets for drug and vaccine development, in particular against viral infection and cancer.

Although mRNA mistranslation can be caused by a variety of mechanisms, tRNA misdecoding and tRNA misacylation are the key drivers. The former is largely attributed to the partially missing tRNAs and excessive wobbling decoding. Consequently, mRNA codons can be coupled to cognate or near-cognate tRNAs at position 3, leading to modulation of translation efficiency and misincorporation [33]. By furthering wobbling at position 1 and 2, mRNA codon can be falsely decoded by “far-cognate” tRNAs. We speculate that if wobbling or superwobbling concurrently occurs at all three positions, especially with regard to the codons decoded by the two box tRNA sets, no functional protein would likely be produced.

The development of high-throughput sequencing and ribosome profiling technologies has greatly advanced our understanding of tRNA decoder [71]. However, proteomic analysis at single molecular level remains technically infeasible. This hampers a detailed characterization of the protein “quasispecies” pool that results from mistranslation. In the future, deciphering single codon–anticodon decoding will help providing more mechanistic insights as to how tRNA decoding relates to translation fidelity.

## References

[1] Crick FH. Codon—anticodon pairing: the wobble hypothesis. Journal of molecular biology. 1966;19(2):548–55. 5969078 10.1016/s0022-2836(66)80022-0

[2] Roy B, Leszyk JD, Mangus DA, Jacobson A. Nonsense suppression by near-cognate tRNAs employs alternative base pairing at codon positions 1 and 3. Proceedings of the National Academy of Sciences. 2015;112(10):3038–43. 10.1073/pnas.1424127112 PMC4364220 25733896

[3] Rozov A, Demeshkina N, Westhof E, Yusupov M, Yusupova G. New Structural Insights into Translational Miscoding. Trends in biochemical sciences. 2016;41(9):798–814. 10.1016/j.tibs.2016.06.001 27372401

[4] Ribas de Pouplana L, Santos MA, Zhu JH, Farabaugh PJ, Javid B. Protein mistranslation: friend or foe? Trends in biochemical sciences. 2014;39(8):355–62. 10.1016/j.tibs.2014.06.002 25023410

[5] Garofalo R, Wohlgemuth I, Pearson M, Lenz C, Urlaub H, Rodnina MV. Broad range of missense error frequencies in cellular proteins. Nucleic Acids Res. Epub 2019 Jan 15. 10.1093/nar/gky1319 30649420 PMC6451103

[6] Mohler K, Ibba M. Translational fidelity and mistranslation in the cellular response to stress. Nat Microbiol. 2017;2:17117 10.1038/nmicrobiol.2017.117 28836574 PMC5697424

[7] Robinson R. Which codon synonym is best? It may depend on what's on the menu. PLoS Biol. 2014;12(12):e1002014 10.1371/journal.pbio.1002014 25489733 PMC4260823

[8] Chan CT, Pang YL, Deng W, Babu IR, Dyavaiah M, Begley TJ, et al Reprogramming of tRNA modifications controls the oxidative stress response by codon-biased translation of proteins. Nature communications. 2012;3:937 10.1038/ncomms1938 22760636 PMC3535174

[9] Cantara WA, Crain PF, Rozenski J, McCloskey JA, Harris KA, Zhang X, et al The RNA Modification Database, RNAMDB: 2011 update. Nucleic Acids Res. 2011;39(Database issue):D195–201. 10.1093/nar/gkq1028 21071406 PMC3013656

[10] Hanson G, Coller J. Codon optimality, bias and usage in translation and mRNA decay. Nature Reviews Molecular Cell Biology. 2017;19:20 10.1038/nrm.2017.91 29018283 PMC6594389

[11] Quax TE, Claassens NJ, Soll D, van der Oost J. Codon Bias as a Means to Fine-Tune Gene Expression. Molecular cell. 2015;59(2):149–61. 10.1016/j.molcel.2015.05.035 26186290 PMC4794256

[12] Endres L, Dedon PC, Begley TJ. Codon-biased translation can be regulated by wobble-base tRNA modification systems during cellular stress responses. RNA biology. 2015;12(6):603–14. 10.1080/15476286.2015.1031947 25892531 PMC4615639

[13] Drummond DA, Wilke CO. The evolutionary consequences of erroneous protein synthesis. Nature reviews Genetics. 2009;10(10):715–24. 10.1038/nrg2662 19763154 PMC2764353

[14] Rubenstein E. Misincorporation of the proline analog azetidine-2-carboxylic acid in the pathogenesis of multiple sclerosis: a hypothesis. Journal of Neuropathology & Experimental Neurology. 2008;67(11):1035–40. 18957898 10.1097/NEN.0b013e31818add4a

[15] Lee JW, Beebe K, Nangle LA, Jang J, Longo-Guess CM, Cook SA, et al Editing-defective tRNA synthetase causes protein misfolding and neurodegeneration. Nature. 2006;443(7107):50–5. 10.1038/nature05096 16906134

[16] Kirino Y, Yasukawa T, Ohta S, Akira S, Ishihara K, Watanabe K, et al Codon-specific translational defect caused by a wobble modification deficiency in mutant tRNA from a human mitochondrial disease. Proceedings of the National Academy of Sciences of the United States of America. 2004;101(42):15070–5. 10.1073/pnas.0405173101 15477592 PMC524061

[17] Rodgers KJ. Non-protein amino acids and neurodegeneration: the enemy within. Exp Neurol. 2014;253:192–6. 10.1016/j.expneurol.2013.12.010 24374297

[18] Santos M, Pereira PM, Varanda AS, Carvalho J, Azevedo M, Mateus DD, et al Codon misreading tRNAs promote tumor growth in mice. RNA biology. 2018;15(6):773–786 10.1080/15476286.2018.1454244 29558247 PMC6152441

[19] Lant JT, Berg MD, Heinemann IU, Brandl CJ, O'Donoghue P. Pathways to disease from natural variations in human cytoplasmic tRNAs. The Journal of biological chemistry. Epub 2019 Jan 14. 10.1074/jbc.REV118.002982 30643023 PMC6462537

[20] Agris PF, Eruysal ER, Narendran A, Vare VYP, Vangaveti S, Ranganathan SV. Celebrating wobble decoding: Half a century and still much is new. RNA biology. 2018;15(4–5):537–553. 10.1080/15476286.2017.1356562 28812932 PMC6103715

[21] Alkatib S, Scharff LB, Rogalski M, Fleischmann TT, Matthes A, Seeger S, et al The contributions of wobbling and superwobbling to the reading of the genetic code. PLoS Genet. 2012;8(11):e1003076 10.1371/journal.pgen.1003076 23166520 PMC3499367

[22] Rogalski M, Karcher D, Bock R. Superwobbling facilitates translation with reduced tRNA sets. Nature structural & molecular biology. 2008;15(2):192–8. 10.1038/nsmb.1370 18193063

[23] Chan PP, Lowe TM. GtRNAdb 2.0: an expanded database of transfer RNA genes identified in complete and draft genomes. Nucleic Acids Research. 2016;44(D1):D184–D9. 10.1093/nar/gkv1309 26673694 PMC4702915

[24] Parisien M, Wang X, Pan T. Diversity of human tRNA genes from the 1000-genomes project. RNA biology. 2013;10(12):1853–67. 10.4161/rna.27361 24448271 PMC3917988

[25] Inagaki Y, Kojima A, Bessho Y, Hori H, Ohama T, Osawa S. Translation of Synonymous Codons in Family Boxes by Mycoplasma capricolum tRNAs with Unmodified Uridine or Adenosine at the First Anticodon Position. Journal of molecular biology. 1995;251(4):486–92. 10.1006/jmbi.1995.0450 7658467

[26] Suzuki T, Nagao A, Suzuki T. Human Mitochondrial tRNAs: Biogenesis, Function, Structural Aspects, and Diseases. Annual Review of Genetics. 2011;45(1):299–329. 10.1146/annurev-genet-110410-132531 21910628

[27] Novoa Eva Maria, Pavon-Eternod Mariana, Pan Tao, RdP Lluís. A Role for tRNA Modifications in Genome Structure and Codon Usage. Cell. 2012;149(1):202–13. 10.1016/j.cell.2012.01.050 22464330

[28] Precup J, Ulrich AK, Roopnarine O, Parker J. Context specific misreading of phenylalanine codons. Molecular and General Genetics MGG. 1989;218(3):397–401. 2685541 10.1007/BF00332401

[29] Parker J, Friesen JD. “Two out of three” codon reading leading to mistranslation in vivo. Molecular and General Genetics MGG. 1980;177(3):439–45. 6768967 10.1007/BF00271482

[30] Schwartz MH, Pan T. Function and origin of mistranslation in distinct cellular contexts. Critical reviews in biochemistry and molecular biology. 2017;52(2):205–19. 10.1080/10409238.2016.1274284 28075177 PMC5548284

[31] Lagerkvist U. "Two out of three": an alternative method for codon reading. Proceedings of the National Academy of Sciences of the United States of America. 1978;75(4):1759–62. 273907 10.1073/pnas.75.4.1759 PMC392419

[32] Ren D, Zhang J, Pritchett R, Liu H, Kyauk J, Luo J, et al Detection and identification of a serine to arginine sequence variant in a therapeutic monoclonal antibody. Journal of Chromatography B. 2011;879(27):2877–84. 10.1016/j.jchromb.2011.08.015 21900054

[33] Blanchet S, Cornu D, Hatin I, Grosjean H, Bertin P, Namy O. Deciphering the reading of the genetic code by near-cognate tRNA. Proceedings of the National Academy of Sciences. 2018;115(12):3018–23. 10.1073/pnas.1715578115 PMC5866558 29507244

[34] Swart EC, Serra V, Petroni G, Nowacki M. Genetic Codes with No Dedicated Stop Codon: Context-Dependent Translation Termination. Cell. 2016;166(3):691–702. 10.1016/j.cell.2016.06.020 27426948 PMC4967479

[35] Bouadloun F, Donner D, Kurland C. Codon-specific missense errors in vivo. The EMBO journal. 1983;2(8):1351–6. 10872330 10.1002/j.1460-2075.1983.tb01591.x PMC555282

[36] Edelmann P, Gallant J. Mistranslation in E. coli. Cell. 1977;10(1):131–7. 138485 10.1016/0092-8674(77)90147-7

[37] Kimsey IJ, Szymanski ES, Zahurancik WJ, Shakya A, Xue Y, Chu CC, et al Dynamic basis for dG*dT misincorporation via tautomerization and ionization. Nature. 2018;554(7691):195–201. 10.1038/nature25487 29420478 PMC5808992

[38] Kimsey IJ, Petzold K, Sathyamoorthy B, Stein ZW, Al-Hashimi HM. Visualizing transient Watson–Crick-like mispairs in DNA and RNA duplexes. Nature. 2015;519:315 10.1038/nature14227 25762137 PMC4547696

[39] Ling J, Reynolds N, Ibba M. Aminoacyl-tRNA synthesis and translational quality control. Annual review of microbiology. 2009;63:61–78. 10.1146/annurev.micro.091208.073210 19379069

[40] Demeshkina N, Jenner L, Westhof E, Yusupov M, Yusupova G. A new understanding of the decoding principle on the ribosome. Nature. 2012;484(7393):256–9. 10.1038/nature10913 22437501

[41] Ruan B, Palioura S, Sabina J, Marvin-Guy L, Kochhar S, LaRossa RA, et al Quality control despite mistranslation caused by an ambiguous genetic code. Proceedings of the National Academy of Sciences. 2008;105(43):16502–7. 10.1073/pnas.0809179105 PMC2575449 18946032

[42] Guo M, Chong YE, Shapiro R, Beebe K, Yang X-L, Schimmel P. Paradox of mistranslation of serine for alanine caused by AlaRS recognition dilemma. Nature. 2009;462:808 10.1038/nature08612 20010690 PMC2799227

[43] Javid B, Sorrentino F, Toosky M, Zheng W, Pinkham JT, Jain N, et al Mycobacterial mistranslation is necessary and sufficient for rifampicin phenotypic resistance. Proceedings of the National Academy of Sciences. 2014;111(3):1132–7. 10.1073/pnas.1317580111 PMC3903211 24395793

[44] Miranda I, Silva-Dias A, Rocha R, Teixeira-Santos R, Coelho C, Gonçalves T, et al Candida albicans CUG mistranslation is a mechanism to create cell surface variation. mBio. 2013;4(4):e00285–13. 10.1128/mBio.00285-13 23800396 PMC3697807

[45] Netzer N, Goodenbour JM, David A, Dittmar KA, Jones RB, Schneider JR, et al Innate immune and chemically triggered oxidative stress modifies translational fidelity. Nature. 2009;462:522 10.1038/nature08576 19940929 PMC2785853

[46] Jenner L, Demeshkina N, Yusupova G, Yusupov M. Structural rearrangements of the ribosome at the tRNA proofreading step. Nature structural & molecular biology. 2010;17(9):1072–8. 10.1038/nsmb.1880 20694005

[47] Ogle JM, Murphy FV IV, Tarry MJ, Ramakrishnan V. Selection of tRNA by the ribosome requires a transition from an open to a closed form. Cell. 2002;111(5):721–32. 12464183 10.1016/s0092-8674(02)01086-3

[48] Kalapis D, Bezerra AR, Farkas Z, Horvath P, Bodi Z, Daraba A, et al Evolution of Robustness to Protein Mistranslation by Accelerated Protein Turnover. PLoS Biol. 2015;13(11):e1002291 10.1371/journal.pbio.1002291 26544557 PMC4636289

[49] Li L, Boniecki MT, Jaffe JD, Imai BS, Yau PM, Luthey-Schulten ZA, et al Naturally occurring aminoacyl-tRNA synthetases editing-domain mutations that cause mistranslation in Mycoplasma parasites. Proceedings of the National Academy of Sciences. 2011;108(23):9378–83. 10.1073/pnas.1016460108 PMC3111296 21606343

[50] Loftfield RB, Vanderjagt D. The frequency of errors in protein biosynthesis. The Biochemical journal. 1972;128(5):1353–6. 4643706 10.1042/bj1281353 PMC1174024

[51] Yadavalli SS, Ibba M. Selection of tRNA charging quality control mechanisms that increase mistranslation of the genetic code. Nucleic Acids Research. 2013;41(2):1104–12. 10.1093/nar/gks1240 23222133 PMC3553970

[52] Liu Y, Satz JS, Vo M-N, Nangle LA, Schimmel P, Ackerman SL. Deficiencies in tRNA synthetase editing activity cause cardioproteinopathy. Proceedings of the National Academy of Sciences. 2014;111(49):17570–5. 10.1073/pnas.1420196111 PMC4267364 25422440

[53] Domingo E, Sheldon J, Perales C. Viral quasispecies evolution. Microbiology and molecular biology reviews: MMBR. 2012;76(2):159–216. 10.1128/MMBR.05023-11 22688811 PMC3372249

[54] Sanjuan R, Nebot MR, Chirico N, Mansky LM, Belshaw R. Viral mutation rates. J Virol. 2010;84(19):9733–48. 10.1128/JVI.00694-10 20660197 PMC2937809

[55] Holmes EC. Error thresholds and the constraints to RNA virus evolution. Trends in microbiology. 2003;11(12):543–6. 14659685 10.1016/j.tim.2003.10.006 PMC7172642

[56] Vignuzzi M, Stone JK, Arnold JJ, Cameron CE, Andino R. Quasispecies diversity determines pathogenesis through cooperative interactions in a viral population. Nature. 2006;439(7074):344–8. 10.1038/nature04388 16327776 PMC1569948

[57] Rai DK, Diaz-San Segundo F, Campagnola G, Keith A, Schafer EA, Kloc A, et al Attenuation of Foot-and-Mouth Disease Virus by Engineered Viral Polymerase Fidelity. Journal of Virology. 2017;91(15):e00081–17. 10.1128/JVI.00081-17 28515297 PMC5651715

[58] Nibert ML. Mitovirus UGA(Trp) codon usage parallels that of host mitochondria. Virology. 2017;507:96–100. 10.1016/j.virol.2017.04.010 28431284 PMC5517309

[59] Ma NJ, Hemez CF, Barber KW, Rinehart J, Isaacs FJ. Organisms with alternative genetic codes resolve unassigned codons via mistranslation and ribosomal rescue. eLife. 2018;7:1–23. 10.7554/eLife.34878 PMC6207430 30375330

[60] Stavrou S, Ross SR. APOBEC3 Proteins in Viral Immunity. Journal of immunology. 2015;195(10):4565–70. 10.4049/jimmunol.1501504 PMC4638160 26546688

[61] Mohammadzadeh N, Follack TB, Love RP, Stewart K, Sanche S, Chelico L. Polymorphisms of the cytidine deaminase APOBEC3F have different HIV-1 restriction efficiencies. Virology. 2019;527:21–31. 10.1016/j.virol.2018.11.004 30448640

[62] Reuman EC, Margeridon-Thermet S, Caudill HB, Liu T, Borroto-Esoda K, Svarovskaia ES, et al A classification model for G-to-A hypermutation in hepatitis B virus ultra-deep pyrosequencing reads. Bioinformatics. 2010;26(23):2929–32. 10.1093/bioinformatics/btq570 20937597 PMC2982158

[63] Topalis D, Gillemot S, Snoeck R, Andrei G. Distribution and effects of amino acid changes in drug-resistant alpha and beta herpesviruses DNA polymerase. Nucleic Acids Res. 2016;44(20):9530–54. 10.1093/nar/gkw875 27694307 PMC5175367

[64] Fearon ER, Vogelstein B. A genetic model for colorectal tumorigenesis. Cell. 1990;61(5):759–67. 2188735 10.1016/0092-8674(90)90186-i

[65] Roberts SA, Gordenin DA. Hypermutation in human cancer genomes: footprints and mechanisms. Nat Rev Cancer. 2014;14(12):786–800. 10.1038/nrc3816 25568919 PMC4280484

[66] Bailey MH, Tokheim C, Porta-Pardo E, Sengupta S, Bertrand D, Weerasinghe A, et al Comprehensive characterization of cancer driver genes and mutations. Cell. 2018;173(2):371–85.e18. 10.1016/j.cell.2018.02.060 29625053 PMC6029450

[67] Tomasetti C, Li L, Vogelstein B. Stem cell divisions, somatic mutations, cancer etiology, and cancer prevention. Science. 2017;355(6331):1330–4. 10.1126/science.aaf9011 28336671 PMC5852673

[68] Jansen AM, van Wezel T, van den Akker BE, Ventayol Garcia M, Ruano D, Tops CM, et al Combined mismatch repair and POLE/POLD1 defects explain unresolved suspected Lynch syndrome cancers. Eur J Hum Genet. 2016;24(7):1089–92. 10.1038/ejhg.2015.252 26648449 PMC5070903

[69] Sulima SO, Hofman IJF, De Keersmaecker K, Dinman JD. How Ribosomes Translate Cancer. Cancer Discov. 2017;7(10):1069–87. 10.1158/2159-8290.CD-17-0550 28923911 PMC5630089

[70] Truitt ML, Ruggero D. New frontiers in translational control of the cancer genome. Nat Rev Cancer. 2016;16(5):288–304. 10.1038/nrc.2016.27 27112207 PMC5491099

[71] Goodarzi H, Nguyen HCB, Zhang S, Dill BD, Molina H, Tavazoie SF. Modulated Expression of Specific tRNAs Drives Gene Expression and Cancer Progression. Cell. 2016;165(6):1416–27. 10.1016/j.cell.2016.05.046 27259150 PMC4915377

[72] Ljungstrom V, Cortese D, Young E, Pandzic T, Mansouri L, Plevova K, et al Whole-exome sequencing in relapsing chronic lymphocytic leukemia: clinical impact of recurrent RPS15 mutations. Blood. 2016;127(8):1007–16. 10.1182/blood-2015-10-674572 26675346 PMC4768426

[73] Starrett GJ, Luengas EM, McCann JL, Ebrahimi D, Temiz NA, Love RP, et al The DNA cytosine deaminase APOBEC3H haplotype I likely contributes to breast and lung cancer mutagenesis. Nature communications. 2016;7:12918 10.1038/ncomms12918 27650891 PMC5036005

[74] Çağlayan M, Wilson SH. Pol μ dGTP mismatch insertion opposite T coupled with ligation reveals promutagenic DNA repair intermediate. Nature communications. 2018;9(1):4213 10.1038/s41467-018-06700-5 30310068 PMC6181931

[75] Li HD, Cuevas I, Zhang M, Lu C, Alam MM, Fu YX, et al Polymerase-mediated ultramutagenesis in mice produces diverse cancers with high mutational load. J Clin Invest. 2018;128(9):4179–91. 10.1172/JCI122095 30124468 PMC6118636

[76] Hartwell LH, Kastan MB. Cell cycle control and cancer. Science. 1994;266(5192):1821–8. 7997877 10.1126/science.7997877

[77] Gaillard H, Garcia-Muse T, Aguilera A. Replication stress and cancer. Nat Rev Cancer. 2015;15(5):276–89. 10.1038/nrc3916 25907220

[78] Ladang A, Rapino F, Heukamp LC, Tharun L, Shostak K, Hermand D, et al Elp3 drives Wnt-dependent tumor initiation and regeneration in the intestine. Journal of Experimental Medicine. 2015;212(12):2057–75. 10.1084/jem.20142288 26527802 PMC4647259

[79] Rapino F, Delaunay S, Zhou Z, Chariot A, Close P. tRNA Modification: Is Cancer Having a Wobble? Trends Cancer. 2017;3(4):249–52. 10.1016/j.trecan.2017.02.004 28718436

[80] Rapino F, Delaunay S, Rambow F, Zhou Z, Tharun L, De Tullio P, et al Codon-specific translation reprogramming promotes resistance to targeted therapy. Nature. 2018;558(7711):605–9. 10.1038/s41586-018-0243-7 29925953

[81] Rapino F, Close P. Wobble uridine tRNA modification: a new vulnerability of refractory melanoma. Mol Cell Oncol. 2018;5(6):e1513725 10.1080/23723556.2018.1513725 30525092 PMC6276846

[82] Kang M, Lu Y, Chen S, Tian F. Harnessing the power of an expanded genetic code toward next-generation biopharmaceuticals. Current Opinion in Chemical Biology. 2018;46:123–9. 10.1016/j.cbpa.2018.07.018 30059835

[83] Kim CH, Axup JY, Dubrovska A, Kazane SA, Hutchins BA, Wold ED, et al Synthesis of bispecific antibodies using genetically encoded unnatural amino acids. Journal of the American Chemical Society. 2012;134(24):9918–21. 10.1021/ja303904e 22642368 PMC4299457

[84] Lu H, Zhou Q, Deshmukh V, Phull H, Ma J, Tardif V, et al Targeting human C-type lectin-like molecule-1 (CLL1) with a bispecific antibody for immunotherapy of acute myeloid leukemia. Angewandte Chemie International Edition. 2014;53(37):9841–5. 10.1002/anie.201405353 25056598 PMC4280064

[85] Yadav M, Jhunjhunwala S, Phung QT, Lupardus P, Tanguay J, Bumbaca S, et al Predicting immunogenic tumour mutations by combining mass spectrometry and exome sequencing. Nature. 2014;515(7528):572–6. 10.1038/nature14001 25428506

[86] Gubin MM, Zhang X, Schuster H, Caron E, Ward JP, Noguchi T, et al Checkpoint blockade cancer immunotherapy targets tumour-specific mutant antigens. Nature. 2014;515(7528):577–81. 10.1038/nature13988 25428507 PMC4279952

[87] Lu J, Bergert M, Walther A, Suter B. Double-sieving-defective aminoacyl-tRNA synthetase causes protein mistranslation and affects cellular physiology and development. Nature communications. 2014;5:5650 10.1038/ncomms6650 25427601 PMC4263187

[88] Hoffman KS, Berg MD, Shilton BH, Brandl CJ, O'Donoghue P. Genetic selection for mistranslation rescues a defective co-chaperone in yeast. Nucleic Acids Res. 2017;45(6):3407–21. 10.1093/nar/gkw1021 27899648 PMC5389508

[89] Raina M, Moghal A, Kano A, Jerums M, Schnier PD, Luo S, et al Reduced amino acid specificity of mammalian tyrosyl-tRNA synthetase is associated with elevated mistranslation of Tyr codons. The Journal of biological chemistry. 2014;289(25):17780–90. 10.1074/jbc.M114.564609 24828507 PMC4067211

[90] Cantara WA, Murphy FV, Demirci H, Agris PF. Expanded use of sense codons is regulated by modified cytidines in tRNA. Proceedings of the National Academy of Sciences. 2013;110(27):10964–9. 10.1073/pnas.1222641110 PMC3704028 23781103

[91] Muhlhausen S, Findeisen P, Plessmann U, Urlaub H, Kollmar M. A novel nuclear genetic code alteration in yeasts and the evolution of codon reassignment in eukaryotes. Genome research. 2016;26(7):945–55. 10.1101/gr.200931.115 27197221 PMC4937558

[92] Wei J, Yewdell JW. Autoimmune T cell recognition of alternative-reading-frame-encoded peptides. Nature medicine. 2017;23(4):409–10 10.1038/nm.4317 28388603

